# Short-Term Response of Sasa Dwarf Bamboo to a Change of Soil Nitrogen Fertility in a Forest Ecosystem in Northern Hokkaido, Japan

**DOI:** 10.3390/plants5020019

**Published:** 2016-04-14

**Authors:** Tsunehiro Watanabe, Karibu Fukuzawa, Hideaki Shibata

**Affiliations:** 1 Field Science Center for Northern Biosphere, Hokkaido University, Kita 9 Nishi 9, Kita-ku, Sapporo, Hokkaido 060-0809, Japan; shiba@fsc.hokudai.ac.jp; 2 Graduate School for Environmental Science, Hokkaido University, Kita10, Nishi 5, Kita-ku, Sapporo, Hokkaido 060-0810, Japan; 3 Field Science Center for Northern Biosphere, Hokkaido University, 483 Otoineppu, Otoineppu, Hokkaido 098-2501, Japan; caribu@fsc.hokudai.ac.jp

**Keywords:** biomass, litter decomposition, nitrogen addition experiment, nitrogen cycling, understory plant

## Abstract

In forest ecosystems, a change of soil nitrogen (N) cycling after disturbance is regulated by various factors. Sasa dwarf bamboo (hereafter referred to as Sasa) is an understory plant that grows thickly on the forest floor in northern Hokkaido, Japan. However, the ecosystem function of Sasa after disturbances in the soil N cycling is not fully understood. The purpose of this study was to determine the short-term response of Sasa to a change of soil N fertility. Biomass, litterfall, litter decomposition, soil N pool, and N leaching from soil were measured in control, and low- (5 g N m^−2^ year^−1^) and high-N (15 g N m^−2^ year^−1^) addition plots. Sasa immobilized much N as the soil N fertility increased. However, the leaf N concentration in aboveground biomass did not increase, suggesting that the N in leaves was maintained because of the increase of leaf biomass. As a result, the decomposition and mineralization rates of the produced litter before and after N addition were comparable among plots, even though the soil inorganic N fertility increased greatly. These results suggest that immediate response of Sasa to an increase of soil inorganic N mitigates the excess N leaching from soil.

## 1. Introduction

Forests supply a variety of ecosystem services, such as the maintenance of water regulation, primary production, carbon (C) sequestration, and biodiversity, which are based on complex balances that occur in forest ecosystems. However, human activities, such as logging, fossil fuel consumption, and fertilizer inputs to farmland, degrade ecosystem services by altering the amount of C dioxide and nitrogen (N) oxide emissions, as well as the material cycling between plants and soil in forest ecosystems [1,2,3,4,5,6]. Notably, anthropogenic disturbances resulting from increased economic development have been predicted to degrade ecosystem services in East Asia [3,4,7,8].

An increase of atmospheric N deposition is one of the main environmental events significantly impacting forest and aquatic ecosystems [3,5,6]. Previous studies have shown that atmospheric N deposition causes an increase of the net primary production of forests [1,9,10], a change of the N concentration and nutrient balances of green and senescent leaves, and C and N mineralization from litter [11,12,13] because N is generally limited in temperate forest ecosystems [14]. An increase of the leaf N concentration and decrease of the soil C/N ratio results in a higher risk of N leaching from soil to streams by enhancing microbial litter decomposition, N mineralization and nitrification [2,12,13,15]. Recently, some studies have reported the impacts of increased N deposition on understory plants [16,17,18,19,20]. For example, the numbers of bryophytes and lichens, which function as N sinks, decreased because of increased atmospheric N deposition, whereas the numbers of herbaceous plants, such as Gramineae, increased [20,21,22]. These results suggest that the initial responses of forest ecosystems to an increase of atmospheric N deposition differ significantly depending on the amount of the original N deposition and the sensitivity of plant species including understory plants to external N. However, the responses of understory plants, especially in the litter dynamics to the change in soil N fertility are not fully understood.

Northern Hokkaido, Japan, has relatively low atmospheric N deposition (*ca.* 2.3 kg N m^−2^ year^−1^) compared with other regions in Japan [23]. Sasa dwarf bamboo (*Sasa senanensis*, Gramineae) (hereafter referred to as Sasa) is a common understory plant that is distributed densely on the forest floor in this region. Sasa, a clonal plant, produces a rhizome and reproduces asexually. It has been indicated that Sasa, having a wide distribution, not only has a negative effect on the natural regeneration of tree plants, but is also important for understanding ecosystem function [24,25,26]. Sasa litterfall accounted for 30% of the total litterfall in a natural forest, and that the litter decomposed more slowly than tree leaf litter because of its high silicate content [26]. It has also been reported that Sasa mitigates increases of soil inorganic N [27,28,29] and N leaching from soil to streams [30,31] because of the rapid annual increment of its aboveground and belowground biomass. These results indicate that Sasa take up the most of increased soil inorganic N after disturbances such as atmospheric N deposition or forest management treatments, suggesting that the change of soil N fertility influence on Sasa litter dynamics. However, it is not known how the Sasa N sink and Sasa litter dynamics change in response to increased N uptake.

Here, we aimed to determine the short-term response of Sasa to a change of soil N fertility. We hypothesized that: (1-1) as soil N fertility increases, the leaf litter N concentration would increase because of increased N uptake by Sasa; (1-2) the decomposition rate of Sasa leaf litter would increase in response to increases of soil N fertility and the litter N concentration; and (2) as soil N fertility increases, the extent of N leaching from soil would be mitigated by the increase of N uptake by Sasa. To test these hypotheses, we conducted a plot-level N addition experiment in a temperate forest in northern Hokkaido, Japan.

## 2. Results

### 2.1. The Response of Sasa and Soil to a Change of Soil N Fertility

N amount effects by N addition were detected for leaf in Sasa aboveground biomass except for current leaf biomass (Table 1). They were significantly higher in the high-N plot than in the other plots in 2009 (*P* ˂ 0.05) (Table 1). In contrast, N amount effects were not found for the N concentration in each organ of the Sasa aboveground biomass (Table S2). Year effect was detected for soil inorganic N (*P* ˂ 0.05) (Table 1).

In the litter decomposition experiment, the initial N concentrations of Sasa leaf litter were not significantly differences among plots in each collected year (Table 2). After one year of decomposition, the dry mass remaining of Sasa leaf litter were not significantly differences among plots in each decomposition period (Table 3). In N mass remaining, the highest value occurred in the high-N plot during the decomposition period from November 2007 to 2008 (*P* ˂ 0.05), and the N mass remaining during the decomposition period from November 2008 to 2009 was significantly higher in low-N plot than in control plot. N amount effects were absent for the litterfall amount and the N concentration in litter fall (Table S4).

### 2.2. Estimation of the N Budget

**Figure 1:**
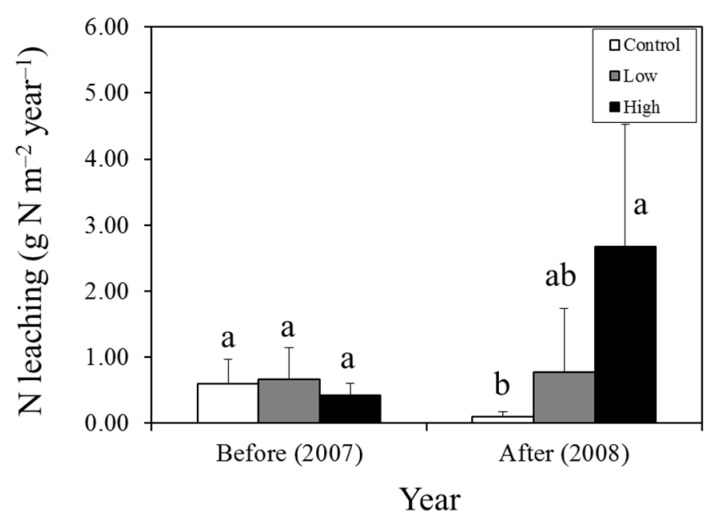
Inorganic N leaching in control, low-, and high-N plots before (2007) and after (2008) N addition. The results of the one-way ANOVA among plots in each year are shown in the graph. The N leaching amount of before and after N addition is calculated as sum of the period from August 2007 to June 2008 and sum of the period from June 2008 to June 2009, respectively. Different lower-case letters indicate significant differences among plots (*P* ˂ 0.05, Tukey’s HSD).

The amount of annual N leaching in the high-N plot after the N addition was significantly higher than that of the other plots (*P* ˂ 0.05) (Figure 1). The amounts of annual N leaching in the low- and high-N plots were 2.2% and 15% of the annual N input, respectively (Figure 1 and Table 4). The amounts of retained N in the whole Sasa and soil system in the low- and high-N plots were 4.89 and 12.8 g N m^−2^ year^−1^, or equivalent to 97.8% and 85% of the annual N input, respectively (Table 4). Regarding the measured N storage in the Sasa and soil system, the total net changes in the low- and high-N plots were −3.56 and 9.14 g N m^−2^, respectively (*i.e.*, sum of the net N storage except the estimated storage in Table 4). In the high-N plot, 57% of the net change of the total measured N storage mass resulted from Sasa aboveground biomass (5.76 g N m^−2^). The estimated amounts of unmeasured N storage (“Estimated N storage” in Table 4) in the Sasa and soil system in the low- and high-N plots were 8.11 and 2.72 g N m^−2^, respectively (Table 4).

## 3. Discussion

### 3.1. Effect of Soil N Fertility Changes on the Leaf N, Biomass and Litter Decomposition

The leaf biomass N increased significantly in the high-N plot compared with the other plots in 2009 (Table 1). However, there was no significant N amount effect on the leaf N concentration in the Sasa aboveground biomass and the litter (Tables S2 and S4), suggesting that the leaf N concentrations in the aboveground biomass and litter did not change, even though the amount of N taken up by the Sasa aboveground biomass increased rapidly in response to the short-term increase of soil N fertility. Previous studies have indicated that after N addition, the leaf N concentration increased by the increase of the amount of N uptake with the decrease of N resorption from green- to senescent-leaves because of the mitigation of the soil N limit [32,33,34,35,36,37,38]. In such case, the N concentration in leaf litter result to increases [32,33,34,35,36,37,38]. However, in this study, the N concentration in leaf litter did not increase (Table S4 and Table 2), suggesting that increase of leaf production (*i.e.*, carbon uptake by photosynthesis) compensated the increase of N concentration by the increase of N uptake in leaf. These mechanisms are known as dilution effects, which have also been reported in CO_2_ fertilization experiments [39,40]. As a result, it was suggested that the litter N concentration after N addition was also maintained by those dilution effects (Table 2).

Results of one-year litter decomposition in both decomposition periods revealed that there were no significant N amount effects on the dry mass remaining during each year (Table 3). This result indicates that the amount of C released by the Sasa leaf litter decomposition does not increase when the soil N fertility increases. The increase of the N concentration in leaf litter and the addition of N directly to the leaf litter usually enhance the decomposition of leaf litter with low lignin content, especially in the initial decomposition phase, which provides much available C for microbes [41]. It has been known that the lignin concentration of the Sasa leaf litter is significantly lower than that of tree leaf litter in our studied region [26]. However, N addition did not affect the C decomposition in the initial decomposition of Sasa leaf litter. It has also been suggested that Sasa litter with a high silicate concentration decomposes more slowly than tree leaf litter [26]. These results suggest that litter with a high concentration of a recalcitrant material, including silicate, responds less to the increase of soil N fertility.

The N mass remaining during litter decomposition was maintained even after the N additions except for the high- and low-N plots in the decomposition period from November 2007 to 2008 and November 2008 to 2009, respectively (Table 3). In a previous study of litter decomposition, leaf litter was often decomposed rapidly in the initial decomposition phase under a high soil N level compared to a low soil N level [41,42]. However, in this study, the N did not release from Sasa leaf litter even though there were sufficient amounts of available N in the surrounding soil. It suggests that Sasa leaf litter has a high resistance for microbial decomposition, even in increased N environments, compared to other plants.

### 3.2. The Response of the Sasa and Soil System to Increased Soil N Fertility

The N leaching from soil increased in the high-N plot after the N addition, whereas there were no significant changes in the low-N plot compared to the control plot (Table 4). These results suggest that the threshold of N retention of the Sasa and soil system to cause N leaching would be between 5 and 15 g N m^−2^ year^−1^. Most excess N was accumulated in Sasa biomass and litter layer even though the short-term increase of soil N fertility was large enough to cause N leaching from soil. Sasa immobilized much of the N, even at the high N input (Table 4). In addition, some N was retained in the estimated N storage, which was not measured directly in this study (Table 4). This unmeasured component would include microbial N retention, N immobilization in decomposing litter (*i.e.*, Oe (partly decomposed and fragmented litter layer) and Oa horizon (well-decomposed litter layer)), dissolved organic N (DON) and denitrification efflux. Previous studies of the ecosystem response to an increase of soil N fertility, such as that resulting from atmospheric N deposition and N addition experiments, have reported that biotic immobilization by microbes and abiotic immobilization by soil organic matter are important N sinks [43,44]. In a site near that of the present study, Shibata, *et al.* [23] reported that most of the added N (5 g N m^−2^ year^−1^) was retained in the watershed during the first year. They also reported that 90% and 50% of the added ^15^NH_4_^+^-N were quickly immobilized by microbes in the Oe/Oa layer and mineral soil, respectively. Those suggested that the estimated N storage in Table 4 would be mostly caused by rapid microbial N immobilization in decomposing litter after the N addition. Further quantification of the unmeasured components including DON and denitrification dynamics is necessary for better understanding.

## 4. Experimental Section

### 4.1. Site Description

**Figure 2:**
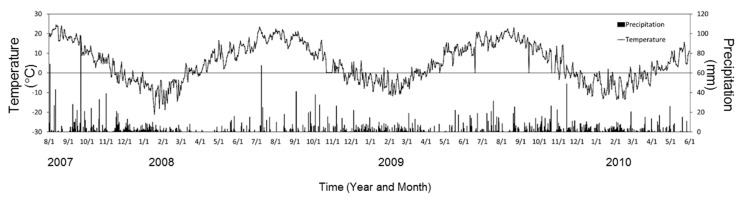
Mean daily precipitation and temperature measured at the Nakagawa station (AMEDAS; Automated Meteorological Data Acquisition System, Japan Meteorological Agency, http://www.data.jma.go.jp) from August 2007 to May 2010.

This study was conducted in a conifer and broadleaf mixed forest in the Nakagawa Experimental Forest in northern Hokkaido, Japan (44°48′N, 142°6′E). The forest floor is covered with understory plants at high density and tall plants (often reaching over 2 m) predominantly consist of Sasa [24,26]. The bedrock is Cretaceous sedimentary rock. The dominant soil is a moderate dry brown forest soil [45] corresponding to Dystric Cambisol [46]. The study area has a cool-temperate climate with heavy snowfall in winter. The mean annual temperature and precipitation from 2008 to 2009 were 6.0 °C and 1038 mm, respectively. The winter season occurs from November to April, and precipitation increases from September to October (Figure 2). The total amount of snow, in terms of water equivalent, is approximately 445 mm, and it accounts for 43% of the total annual precipitation. The maximum snowpack depth is approximately 2 m [23,24,26]. The atmospheric N deposition in this area is about 2.3 kg N ha^−1^ year^−1^ [23].

### 4.2. N Addition Experiment

The N addition experiment was conducted on a flat ridge (about 190 m above sea level) that is dominated by Sasa. The experimental period was from August 2007 to May 2010. We established three experimental plots of N addition: control, low-N (5 g N m^−2^ year^−1^), and high-N (15 g N m^−2^ year^−1^) plots. The annual N addition in the low- and high-N plots corresponds to 22- and 65-times the atmospheric N deposition in this area, respectively. The amount of N addition in low-N plot was decided by the maximum N deposition in urban area in Japan [47]. The Sasa aboveground connects with the rhizome, through which N is transferred [48,49]. Therefore, N addition and control plots should have enough distance to avoid N transfer through rhizome between plots with and without N addition. We established 180 m^2^ (60 m long × 3 m wide) plots for control and N addition. The average distance between the plots was 20 m. We also established four square subplots for replications in each plot. The plant species in each plot were mostly dominated by Sasa, and there were no trees in each subplot, although some canopy tree existed surrounding each subplot. The dominant tree species in the near location in this study were described in [26]. We surveyed Sasa biomass, litterfall, litter decomposition, soil N pool, and N leaching from soil in each subplot. The area of each subplot was 4 m^2^, and each subplot was surrounded by a 50-cm buffer zone. We began to add N in June 2008. The forest floor of the control subplots were sprayed, using a hand sprayer, with 2 L of deionized water, and the forest floor of the N addition subplots were sprayed with 2 L of a NH_4_NO_3_ solution from June to October in 2008 and 2009 (N was applied five times per year). The concentration of the NH_4_NO_3_ solution sprayed on the low- and high-N plots was adjusted to 2000 mg L^−1^ and 6000 mg L^−1^, respectively. The application time was 3 h. The application amount was determined by adjusting the amount of the NH_4_NO_3_ solution to the 3-h average precipitation from June 2007 to October 2007.

### 4.3. Aboveground Biomass

We established quadrats (50 cm × 50 cm) at three corners in each subplot. We collected aboveground Sasa biomass from the surface in each quadrat in late September of 2007, 2008, and 2009 when Sasa biomass is almost at its peak. The collected Sasa biomass was divided into current- and previous-year leaves and culms, and their numbers were measured. The samples were brought to the laboratory, dried for 72 h at 70 °C, and weighed.

### 4.4. Litterfall

Sasa litterfall was measured from September 2007 to May 2010. The litterfall was collected using plastic box litter traps (34 cm long × 49 cm wide × 15 cm high) covered with a nylon net (1-mm mesh). The litter traps were placed in the center of each subplot. Details regarding the method of setting the litter traps and the treatment of litterfall in winter are the same as [26]. Collected litterfall was divided into Sasa leaves and Sasa culms, and the dry weight of each litter was measured after drying for 72 h at 70 °C.

### 4.5. Litter Decomposition Experiment

Sasa leaf litter decomposition was measured by the litterbag method [26]. The produced litter in each subplot was used for litter decomposition experiment in each subplot. We used produced leaf litter before (2007) and after (2008) N addition. The decomposition periods were from November 2007 to 2008 in the produced leaf litter before (October 2007) and from November 2008 to 2009 in the produced leaf litter after (October 2008). We also collected some dead Sasa leaves from the standing stock in each subplot to obtain the required amount for the litterbag experiment. Collected litter samples were air dried and mixed well. Each litterbag contained 3 g of air-dried leaf litter. Litterbags were placed *in situ* in November in each year. External N was sprayed to the litterbags in the period from June to October in each year. There were three litterbag replicates in each subplot. The details of the size of the litterbags and the method of setting the litterbags in the field are the same as [26]. We collected a total of 36 litterbags (three N addition plots × four subplots × three replicates) in each year. The collected litterbags were transferred to the laboratory. After the soil and plants around the bag were carefully removed, the litter in each litterbag was dried for 72 h at 70 °C and weighed.

### 4.6. Soil and Belowground Biomass

Soil from a 0 to 30 cm depth was collected using a soil auger (4.2-cm inner diameter) at the same place used for sampling the aboveground biomass in late September of 2007, 2008 and 2009. The collected soil core was divided into 0–15 cm and 15–30 cm depths. Roots and coarse fragments were removed from each soil depth. The dry weight of the soil was measured after drying for 48 h at 105 °C to determine the soil water content. The belowground biomass at each depth was divided into fine (<2 mm) and large (>2 mm) roots, and their dry weights were measured after drying for 72 h at 70 °C.

### 4.7. Inorganic N Leaching

The amount of leached inorganic N was measured using an ion-exchange resin (IER) bag [50]. We placed 100 g of IER into the nylon stocking bag and placed it in a container (8.4 cm diameter, 2.7 cm high) made of chloroethylene. The container was buried at a 30 cm soil depth in each subplot. The installation periods were from August 2007 to November 2007, November 2007 to June 2008, June 2008 to November 2008, and November 2008 to June 2009. The collected IER bags were transferred to the laboratory. After the soil and plants around the IER bags were carefully removed, the IER subsamples were dried for 48 h at 105 °C and weighed to determine the water content of the IER. N leaching flux (g N m^−2^ year^−1^) from soil was quantified using the following Equation:

N Leaching = N in IER/Area

where *N in IER* indicates N adsorbed in the IER (gN) and *Area* is the collecting areas of the IER container (8.4 cm diameter).

### 4.8. Chemical Analysis

Dried plant samples were ground using a ball mill and analyzed for total C and N concentrations using a CHNS/O analyzer (PE2400 II; PerkinElmer Co., Ltd., Waltham, MA, USA).

Ten grams of fresh soil was extracted using 2 M KCl (50 mL). The extracts were shaken for 1 h and filtered (Advantec No. 5B, Tokyo, Japan). The concentrations of NH_4_^+^ and NO_3_^−^ in the extracted solution were analyzed using an auto analyzer (AACS-4, BL-TEC Inc., Osaka, Japan).

Four grams of fresh resin was extracted using 1 M KCl (200 mL). The extracts were shaken for 1 h and filtered. After filtration, the IER was extracted again. The extracted solution was mixed well, and the concentrations of NH_4_^+^ and NO_3_^−^ were analyzed using an auto analyzer.

### 4.9. Statistical Analysis

To analyze the differences among plots before N addition, all the data before N addition were tested using one-way analysis of variance (ANOVA), followed by Tukey’s honestly significant difference (HSD) test when the effect was significant (*P* ˂ 0.05). To analyze the effect of year, N amount, and year × N amount, all the data after N addition were tested using two-way analysis of variance (ANOVA), followed by Tukey’s honestly significant difference (HSD) test; the interaction effect was considered to be significant at *P* ˂ 0.05. When the effects were significant separately, the data were tested using one-way ANOVA for N amount effect, followed by Tukey’s HSD test when the effect was significant (*P* ˂ 0.05) and a *t*-test when the year effect was significant (*P* ˂ 0.05). For decomposition experiment and N leaching, we tested the differences among plots in each year using one-way ANOVA. The statistical analyses were performed using R software (R version 2.15.1, Free Software Foundation, Inc. Boston, USA. The R Foundation for Statistical Computing http://www.r-project.org/).

### 4.10. Estimation of the N Budget

To clarify a quantitative alteration by N addition, net changes in each data (N input, N leaching, aboveground and belowground biomass N, litterfall N, N mass remaining, and soil inorganic N) were calculated using Equation (1). N retention was calculated using Equation (2). Based on the N budget, we estimated the N storage, which we was not measured directly (Equation (3)).

Net change = data after N addition – data before N addition
(1)

N retention = N input – N leaching
(2)

Estimated N storage = N retention – total measured N storage
(3)

where estimated N storage includes the unmeasured N component, such as microbial N retention, N immobilization in decomposing litter (*i.e.*, Oe and Oa horizon), dissolved organic N and denitrification efflux. This budget was developed for one year before and after the N addition.

## 5. Conclusions

This study targeted Sasa, which thickly grows on the forest floor in northern Hokkaido, and it clarified the short-term response to the increase of soil N fertility. We found that N immobilization by Sasa increased when the external amount of N was increased at 15 g N m^−2^ year^−1^. As a result, N leaching from soil was minor contribution in the N budget, although the N leaching amount after N addition was approximately 3 g N m^−2^ year^−1^ that was equivalent to more than 10 times the current rate of atmospheric N deposition (*ca.* 0.23 g N m^−2^ year^−1^) in this region. Additionally, the N concentration in the aboveground biomass and leaf litter did not increase because the N concentration in the aboveground biomass was diluted by the increase of the leaf biomass. The decomposition and mineralization rates of Sasa leaf litter were not stimulated by N addition, although it has been known that the increase of litter N concentration and soil N fertility usually enhance the microbial decomposition. These results suggest that Sasa can mitigate the changes of short-term soil N dynamics and the N leaching from soil even at higher N inputs (15 g m^−2^ year^−1^) because Sasa immediately takes up the increasingly available soil N after disturbances. This study also suggests that organic layer is an important sink for external N at the low level N input such as 5 g m^−2^ year^−1^. Additionally, the results suggest that the N retention capacity in the Sasa and soil system is significantly higher than the current rate of atmospheric N deposition (*ca.* 0.23 g N m^−2^ year^−1^). 

Previous reports indicate that tree cutting has negative effects on ecosystem structure and functioning because of the rapid changes in N cycling including the stimulation of N mineralization, nitrification and leaching [51,52,53]. This study indicated that a forest ecosystem with dense Sasa is more resilient to rapidly changing soil N availability after some natural and anthropogenic disturbances. The characteristics of Sasa, such as being a clonal plant with persistent litter, contribute to the mechanisms of mitigating dramatic changes in soil N dynamics. Therefore, Sasa is an important understory plant for the practice of ecosystem management including an assessment of ecosystem function in this region.

## Figures and Tables

**Table 1:** Number, biomass, and biomass N in leaf (Total, current, and previous) in Sasa aboveground and soil inorganic N in control, and low- and high-N plots after N addition with *P* values of two-way ANOVA (year, N amount, and the interaction).

Year	Control Plot			Low–N Plot			High–N Plot			Two–Way ANOVA	*P* Value
	Mean	SD		Mean	SD		Mean	SD			
Total leaf number (number m^–2^)											
1 year After	263	143	aA ^(1)^	289	264	aA	619	372	aA	Year (Y)	ns
2 year After	300	197	bA	275	116	bA	697	209	aA	Treatment (T)	˂0.01
										Interaction T × Y	ns
Total leaf biomass (g DW m^–2^)											
1 year After	321	137	aA	297	205	aA	596	316	aA	Year (Y)	ns
2 year After	218	108	bA	205	67	bA	512	167	aA	Treatment (T)	˂0.01
										Interaction T × Y	ns
Total leaf biomass N (g N m^–2^)											
1 year After	6.1	2.7	aA	5.5	4.3	aA	11.4	6.2	aA	Year (Y)	ns
2 year After	4.5	1.8	bA	3.9	1.3	bA	10.8	3.6	aA	Treatment (T)	˂0.01
										Interaction T × Y	ns
Current leaf number (number m^–2^)											
1 year After	71	47	aA	107	148	aA	165	103	aA	Year (Y)	ns
2 year After	155	64	bA	115	83	bA	300	90	aA	Treatment (T)	˂0.05
										Interaction T × Y	ns
Current leaf biomass (g DW m^–2^)											
1 year After	90	44	aA	87	113	aA	129	87	aA	Year (Y)	ns
2 year After	107	19	aA	80	56	aA	150	72	aA	Treatment (T)	0.0579
										Interaction T × Y	ns
Current leaf biomass N (g N m^–2^)											
1 year After	1.9	1.0	aA	1.8	2.4	aA	3.4	2.1	aA	Year (Y)	ns
2 year After	2.6	0.3	bA	1.9	1.3	bA	4.5	1.4	aA	Treatment (T)	˂0.05
										Interaction T × Y	ns
Previous leaf number (number m^–2^)											
1 year After	192	100	aA	182	116	aA	454	271	aA	Year (Y)	ns
2 year After	145	140	bA	160	53	bA	397	136	aA	Treatment (T)	˂0.01
										Interaction T × Y	ns
Previous leaf biomass (g DW m^–2^)											
1 year After	231	96	aA	210	98	aA	446	230	aA	Year (Y)	ns
2 year After	111	98	bA	125	42	bA	315	103	aA	Treatment (T)	˂0.01
										Interaction T × Y	ns
Previous leaf biomass N (g N m^–2^)											
1 year After	4.1	1.8	aA	3.6	2.1	aA	8.0	4.1	aA	Year (Y)	ns
2 year After	1.9	1.7	bA	2.1	0.6	bA	6.3	2.4	aA	Treatment (T)	˂0.01
										Interaction T × Y	ns
Soil inorganic N (NH_4_^+^ and NO_3_^–^) amount (g m^–2^)											
1 year After	5.43	1.48	aA	3.92	0.91	aA	3.94	0.73	aA	Year (Y)	˂0.01
2 year After	3.17	1.33	aA	2.06	0.53	aB	3.17	0.83	aA	Treatment (T)	ns
										Interaction T × Y	ns

September 2008 and 2009 are referred to as 1 year and 2 year, respectively. ^(1)^ Different lower-case letters indicate significant differences among plots in each year (*P* ˂ 0.05, Tukey’s HSD); different capital letters indicate significant differences between years in each plot (*P* ˂ 0.05, *t*-test) in each category; and ns means no significant differences.

**Table 2:** Initial leaf litter N concentration for litter decomposition experiment before and after N addition in control, and low- and high-N plots with *P* values of the one-way ANOVA.

Collected Year of Used Litter	Control		Low–N		High–N		One–Way ANOVA *P* Value
	Mean	SD	Mean	SD	Mean	SD	
Initial leaf litter N concentration (mg g^–1^)							
October (2007)	10.4	1.8	11.2	3.0	8.6	2.2	ns
October (2008)	12.1	1.7	11.2	1.3	12.4	1.1	ns

ns means no significant difference.

**Table 3:** Dry mass and N mass remaining after one-year decomposition in each decomposition period in control, and low- and high-N plots with *P* values of the one-way ANOVA.

Collected Year of Used Litter	Decomposition Period	Control			Low-N			High-N			One-Way ANOVA *P* Value
		Mean	SD		Mean	SD		Mean	SD		
Dry mass remaining (%)											
October (2007)	November 2007 to 2008	74.1	4.5	a ^(1)^	75.9	3.0	a	74.2	3.3	a	ns
October (2008)	November 2008 to 2009	72.1	3.3	a	71.5	4.3	a	69.7	4.4	a	ns
N mass remaining (%)											
October (2007)	November 2007 to 2008	108.8	22.1	b	106.5	28.9	b	140.6	39.3	a	˂0.05
October (2008)	November 2008 to 2009	82.9	17.3	b	103.6	20.0	a	92.9	11.4	ab	˂0.05

^(1)^ Different lower-case letters indicate significant differences among plots in each decomposition period (*P* ˂ 0.05, Tukey's HSD); and ns means no significant difference.

**Table 4:** N input–output, measured N storage, and estimated N storage in each year and their net change in low- and high-N plots.

Component	Low-N Plot		Net Change (g N m^–2^)	High-N Plot		Net Change (g N m^−2^)
	Before	After		Before	After	
N input–output (g N m^−2^ year^−1^)						
N input	0	5	5	0	15	15
N leaching	0.66	0.77	0.11	0.42	2.67	2.25
N retention			4.89			12.75
N storage (g N m^−2^)						
Aboveground biomass N	15.68	9.26	−6.42	13.36	19.12	5.76
Belowground biomass N	4.59	7.29	2.71	5.54	8.12	2.58
N mass remaining in litter	0.85	1.18	0.33	1.37	1.73	0.36
Soil inorganic N	4.10	3.92	−0.18	3.50	3.94	0.44
Estimated N storage			8.11			2.72

N retention is calculated by subtracting N leaching from N input in net change in each plot (Equation (2)); N mass remaining in litter = Litterfall N amount (g N m^−2^ year^−1^) × N mass remaining (%) after one-year decomposition/100. Estimated N storage is estimated by subtracting total in measured N storage from N retention (Equation 3).

## Supplementary Materials

Supplementary Materials can be found at www.mdpi.com/2223-7747/5/2/19/s1.
