# Supplementary material for: Short-Term Response of Sasa Dwarf Bamboo to a Change of Soil Nitrogen Fertility in a Forest Ecosystem in Northern Hokkaido, Japan
Source: Plants (Basel). 2016 Apr 14;5(2):19. doi: 10.3390/plants5020019 (PMC4931399; doi:10.3390/plants5020019)
Supplement: Supplementary file 1 [file plants-05-00019-s001.pdf]

Supplementary Materials: Short-Term Response of Sasa Dwarf Bamboo to a Change of Soil Nitrogen Fertility in a Forest Ecosystem in Northern Hokkaido, Japan

Tsunehiro Watanabe [1,2,*], Karibu Fukuzawa [3,†] and Hideaki Shibata [1,†]

Table S1. Data of aboveground- and belowground-biomass, and their N concentrations in control, low-, and high-N plots before-N addition with the *P* values of the one-way ANOVA among plots in each category.

Category	Control plot		Low–N plot		High–N plot		One–way ANOVA
	mean	SD	mean	SD	mean	SD	*P* value
Total aboveground biomass (g DW m–2)	1733	466	1931	863	1607	322	ns
Total aboveground biomass N (g N m–2)	14.9	3.8	15.7	7.0	13.4	2.6	ns
Total leaf number (number m^{-2})	455	167	492	248	416	91	ns
Total leaf biomass (g DW m^{-2})	427	69	432	169	381	85	ns
Total leaf biomass N (g N m^{-2})	8.6	1.8	8.6	3.6	6.8	1.2	ns
Current leaf number (number m^{-2})	195	82	168	77	138	31	ns
Current leaf biomass (g DW m^{-2})	167	41	147	51	80	26	ns
Current leaf biomass N (g N m^{-2})	3.9	0.7	3.5	1.3	2.6	0.4	ns
Previous leaf number (number m^{-2})	260	110	324	173	278	87	ns
Previous leaf biomass (g DW m^{-2})	261	45	285	118	251	71	ns
Previous leaf biomass N (g N m^{-2})	4.7	1.4	5.2	2.3	4.1	1.0	ns
Total culm number (number m^{-2})	157	73	203	81	145	27	ns
Total culm biomass (g DW m^{-2})	1305	409	1499	701	1226	257	ns
Total culm biomass N (g N m^{-2})	6.3	2.2	7.1	3.5	6.6	1.8	ns
Current culm number (number m^{-2})	45	13	40	19	36	13	ns
Current culm biomass (g DW m^{-2})	266	96	205	98	200	87	ns
Current culm biomass N (g N m^{-2})	2.0	0.5	1.6	0.9	1.5	0.7	ns
Previous culm number (number m^{-2})	112	70	163	62	109	29	ns
Previous culm biomass (g DW m^{-2})	1040	423	1293	618	1025	253	ns
Previous culm biomass N (g N m^{-2})	4.3	2.7	5.4	2.7	5.0	1.6	ns
Current leaf N concentration (mg g^{-1})	23.7	2.2	23.4	1.8	20.8	1.0	ns
Current culm N concentration (mg g^{-1})	7.9	1.8	7.3	1.5	7.6	0.5	ns
Previous leaf N concentration (mg g^{-1})	17.1	2.0	17.6	2.1	16.6	1.4	ns
Previous culm N concentration (mg g^{-1})	4.4	1.3	4.6	0.4	5.1	1.2	ns
Total belowground biomass (g DW m^{-2})	322	185	545	308	688	146	ns
Total belowground biomass N (g N m^{-2})	3.2	2.0	4.6	2.6	5.5	1.1	ns
Fine root N concentration in 0-15 cm depth (mg g^{-1})	10.6	1.8	9.9	0.7	9.9	0.9	ns
Fine root N concentration in 15-30 cm depth (mg g^{-1})	9.7	2.8	8.2	0.8	8.5	0.4	ns
Soil inorganic N (NH_4^+ and NO_3^-) amount (g m^{-2})	5.11	0.95	4.10	1.39	3.50	1.18	ns

ns means no significant.

Table S2. Data of aboveground- and belowground-biomass, and their N concentrations in control, low-, and high-N plots after N addition with the *P* values of the two-way ANOVA (year, N amount, and the interaction).

Year	Control plot			Low–N plot			High–N plot			Two–way ANOVA	*P* value
	mean	SD		mean	SD		mean	SD			
Total aboveground biomass (g DW m^{-2})											
1year After	1290	556	aA[(1)]	1257	633	aA	2060	1238	aA	Year (Y)	ns
2year After	979	676	abA	981	281	bA	2325	861	aA	Treatment (T)	<0.05
										Interaction T × Y	ns
Total aboveground biomass N (g N m^{-2})											
1year After	10.2	3.5	aA	9.3	5.2	aA	19.1	10.9	aA	Year (Y)	ns
2year After	11.2	6.9	abA	8.5	2.8	bA	21.4	6.4	aA	Treatment (T)	<0.01
										Interaction T × Y	ns
Total culm number (number m^{-2})											
1year After	101	45	aA	114	61	aA	178	103	aA	Year (Y)	ns
2year After	84	59	bA	90	39	bA	238	49	aA	Treatment (T)	<0.01
										Interaction T × Y	ns
Total culm biomass (g DW m^{-2})											
1year After	969	423	aA	960	446	aA	1464	927	aA	Year (Y)	ns
2year After	761	569	aA	776	228	aA	1814	697	aA	Treatment (T)	ns
										Interaction T × Y	ns
Total culm biomass N (g N m^{-2})											
1year After	4.1	0.9	aA	3.8	1.0	aA	7.7	4.9	aA	Year (Y)	ns
2year After	6.8	5.2	aA	4.6	1.6	aA	10.6	3.0	aA	Treatment (T)	ns
										Interaction T × Y	ns
Current culm number (number m^{-2})											
1year After	15	11	aA	20	28	aA	32	20	aA	Year (Y)	ns
2year After	32	17	abA	19	15	bA	59	16	aA	Treatment (T)	<0.05
										Interaction T × Y	ns
Current culm biomass (g DW m^{-2})											
1year After	85	43	aA	71	87	aA	140	93	aA	Year (Y)	ns
2year After	302	175	aA	220	174	aA	343	262	aA	Treatment (T)	ns
										Interaction T × Y	ns
Current culm biomass N (g N m^{-2})											
1year After	0.6	0.3	aB	0.4	0.5	aA	1.0	0.7	aA	Year (Y)	<0.05
2year After	3.5	2.4	aA	1.9	1.4	aA	3.0	1.5	aA	Treatment (T)	ns
										Interaction T × Y	ns
Previous culm number (number m^{-2})											
1year After	86	35	aA	94	37	aA	146	83	aA	Year (Y)	ns
2year After	52	45	bA	71	32	bA	179	47	aA	Treatment (T)	<0.01
										Interaction T × Y	ns
Previous culm biomass (g DW m^{-2})											
1year After	883	386	aA	889	389	aA	1324	836	aA	Year (Y)	ns
2year After	458	397	bA	556	183	bA	1471	477	aA	Treatment (T)	<0.05
										Interaction T × Y	ns

Table S2. *Cont.*

Year	Control plot			Low–N plot			High–N plot			Two–way ANOVA	P value
	mean	SD		mean	SD		mean	SD			
Previous culm biomass N (g N m^{-2})											
1year After	3.5	0.7	aA	3.4	0.7	aA	6.7	4.2	aA	Year (Y)	ns
2year After	3.3	2.8	bA	2.7	0.6	bA	7.6	1.6	aA	Treatment (T)	<0.01
										Interaction T × Y	ns
Current leaf N concentration (mg g^{-1})											
1year After	21.6	1.8	aA	21.1	1.9	aA	22.4	0.9	aA	Year (Y)	ns
2year After	23.9	1.7	aA	23.2	1.0	aA	23.4	1.4	aA	Treatment (T)	ns
										Interaction T × Y	ns
Current culm N concentration (mg g^{-1})											
1year After	6.8	0.8	aB	5.4	0.1	aB	7.4	0.6	aB	Year (Y)	<0.05
2year After	10.6	2.5	aA	8.8	1.9	aA	9.5	2.1	aA	Treatment (T)	ns
										Interaction T × Y	<0.05
Previous leaf N concentration (mg g^{-1})											
1year After	17.2	0.9	aA	16.7	2.7	aA	17.5	1.3	aA	Year (Y)	ns
2year After	17.1	0.0	aA	16.7	1.0	aA	19.7	1.5	aA	Treatment (T)	ns
										Interaction T × Y	ns
Previous culm N concentration (mg g^{-1})											
1year After	4.6	1.1	aB	4.7	0.6	aA	5.4	0.8	aA	Year (Y)	<0.05
2year After	7.2	0.2	aA	5.2	1.0	aA	5.4	0.7	aA	Treatment (T)	ns
										Interaction T × Y	ns
Total belowground biomass (g DW m^{-2})											
1year After	1480	1123	aA	1269	708	aA	990	645	aA	Year (Y)	ns
2year After	681	330	aA	498	245	aA	769	650	aA	Treatment (T)	ns
										Interaction T × Y	ns
Total belowground biomass N (g N m^{-2})											
1year After	10.4	6.7	aA	7.3	3.9	aA	8.1	5.7	aA	Year (Y)	ns
2year After	7.3	2.8	aA	4.6	1.9	aA	5.6	3.5	aA	Treatment (T)	ns
										Interaction T × Y	ns
Fine root N concentration in 0-15 cm depth (mg g^{-1})											
1year After	10.2	1.5	aA	9.4	2.8	aA	9.2	1.0	aA	Year (Y)	ns
2year After	12.6	2.0	aA	12.1	3.0	aA	9.3	0.7	aA	Treatment (T)	ns
										Interaction T × Y	ns
Fine root N concentration in 15-30 cm depth (mg g^{-1})											
1year After	6.4	1.6	aA	7.6	0.4	aA	6.5	0.7	aB	Year (Y)	<0.05
2year After	8.7	1.3	aA	7.6	1.2	aA	8.2	1.4	aA	Treatment (T)	ns
										Interaction T × Y	ns

1year and 2year after means September 2008 and 2009, respectively; (1) Different lower-case letters indicate significant differences among plots in each year ($P < 0.05$, Tukey's HSD). Different capital letters indicate significant differences between years in each plot ($P < 0.05$, *t*-test) in each category; ns means no significant.

Table S3. Total littefall amount, N amount, and the N concentrations in leaf and culm litterfall from August 2007 to June 2008 in control, low-, and high-N plots before N addition with *P* values of the one-way ANOVA among plots in each category.

**Category**	**Control plot**			**Low-N plot**			**High-N plot**			**One-way ANOVA**
	mean	SD		mean	SD		mean	SD		*P* value
Total litterfall amount (g m^{-2})	98	85	a	89	27	a	113	62	a	ns
Total litterfall N amount (g m^{-2})	1.1	1.1	a	0.8	0.2	a	1.0	0.4	a	ns
Leaf litterfall N concentration (mg g^{-1})	11.8	2.6	a	12.1	1.8	a	11.4	1.8	a	ns
Culm litterfall N concentration (mg g^{-1})	8.2	2.5	a	4.8	0.7	b	5.2	1.3	ab	<0.05

ns means no significant; Leaf and culm litterfall N concentration shows the weighted-mean N concentration that is calculated by dividing the sum of N mass of the monthly litterfall by total litterfall mass.

Table S4. Total littefall amount, N amount, and the N concentrations in leaf and culm litterfall in control, low-, and high-N plots after N addition with *P* values of the two-way ANOVA.

**Year**	**Control plot**			**Low-N plot**			**High-N plot**				
	**mean**	**SD**		**mean**	**SD**		**mean**	**SD**		**Two-way ANOVA**	***P* value**
				Total litterfall amount (g m^{-2})							
1year After	124	100	aA[(1)]	152	61	aA	184	34	aA	Year (Y)	ns
2year After	148	30	aA	133	86	aA	131	36	aA	Treatment (T)	ns
										Interaction T × Y	ns
				Total litterfall N amount (g N m^{-2})							
1year After	1.1	0.7	aA	1.1	0.4	aA	1.9	0.4	aA	Year (Y)	ns
2year After	1.3	0.2	aA	1.2	1.0	aA	1.0	0.1	aA	Treatment (T)	ns
										Interaction T × Y	ns
				Leaf litterfall N concentration (mg g^{-1})							
1year After	13.1	1.4	aA	14.4	0.5	aA	14.3	2.4	aA	Year (Y)	ns
2year After	11.9	1.0	aA	12.4	2.4	aA	11.9	1.6	aA	Treatment (T)	ns
										Interaction T × Y	ns
				Culm litterfall N concentration (mg g^{-1})							
1year After	4.7	1.2	aA	3.7	0.9	aA	4.8	0.8	aA	Year (Y)	ns
2year After	3.9	0.5	aA	3.9	1.3	aA	4.8	1.2	aA	Treatment (T)	ns
										Interaction T × Y	ns

1year and 2 year after means June 2008–2009 and June 2009–2010, respectively; [(1)] Different lower-case letters indicate significant differences among plots in each year ($P < 0.05$, Tukey's HSD). Different capital letters indicate significant differences between years in each plot ($P < 0.05$, *t*-test) in each category; ns means no significant; Leaf and culm litterfall N concentration shows the weighted-mean N concentration that is calculated by dividing the sum of N mass of the monthly litterfall by total litterfall mass.
